# Temporal dynamics of socioeconomic inequalities in COVID-19 – a scoping review: Florian Beese

**DOI:** 10.1093/eurpub/ckac130.013

**Published:** 2022-10-25

**Authors:** F Beese, L Wollgast, J Waldhauer, J Hoebel, B Wachtler

**Affiliations:** Division for Social Determinants of Health, Robert Koch-Institute, Berlin, Germany

## Abstract

**Background:**

International evidence shows a socially unequal burden of SARS-CoV-2 infections and COVID-19 outcomes across different socioeconomic groups. However, less is known about the temporal dynamics of socioeconomic inequalities in COVID-19 throughout the pandemic. We conducted a scoping review to systematically map and synthesize the available evidence.

**Methods:**

We conducted a systematic literature search in Embase and Scopus with pre-defined eligibility criteria, including empirical research from 1 January 2020 to 24 August 2021. Additionally, several journals and the reference lists of all included articles were hand-searched to identify relevant studies. We used a standardized charting approach to extract relevant content and narratively synthesized the included evidence. The study follows the PRISMA guidelines for scoping reviews.

**Results:**

From 8,011 identified records, we finally included 46 articles in the analysis. 50.0% of all included studies were conducted in the United States. The majority of all studies analyzed surveillance data (n = 44) and used area-based socioeconomic indicators on an ecological level (92.5%). The study results show temporal dynamics in COVID-19 inequalities, frequently initiated through higher outcome rates in more affluent populations and subsequent crossover dynamics to higher rates in more deprived populations (41.9%). Furthermore, 91.4% of the analyses show maintaining or worsening social inequalities in health with ongoing pandemic progression, which hit the most deprived populations the hardest.

**Conclusions:**

The results show worsening social inequalities in COVID-19 over the course of the pandemic, with the most disadvantaged populations most affected during its progression. Targeted prevention and interventions, such as low-threshold testing and vaccination programs, infection protection for precarious jobs or living conditions, and targeted information are crucial to face socioeconomic inequalities throughout pandemics.

**Key messages:**

- Socioeconomic inequalities in COVID-19 show temporal dynamics over the course of the pandemic.
- Socioeconomically deprived populations had higher burdens of COVID-19 outcomes, especially in more advanced stages of the pandemic.

